# Impact of social disadvantages in the presence of diabetes at old age

**DOI:** 10.1186/s12889-019-7348-2

**Published:** 2019-07-29

**Authors:** María Fernanda Carrillo-Vega, Cidronio Albavera-Hernández, Ricardo Ramírez-Aldana, Carmen García-Peña

**Affiliations:** 1 National Institute of Geriatrics, México City, Mexico; 2 Mexican Institute of Social Security, México City, Morelos Mexico; 3 Head of the Research Division, National Institute of Geriatrics, Periférico Sur No. 2767, Col. San Jeronimo Lidice, Del. La Magdalena Contreras, D.F. 10200 México City, Mexico

**Keywords:** Adverse childhood experiences, Social deprivation, Diabetes, Older people

## Abstract

**Background:**

Social disadvantages that start during childhood and continue into the later stages in life may be linked to the presence of diabetes during adulthood. *Objective*. To analyze whether the presence of social disadvantages in childhood and in the present affects the presence of diabetes in older adults.

**Methods:**

The present study was based on longitudinal data from the third and fourth Mexican Health and Aging Study (MHAS) waves (2012 and 2015). Data on diabetes diagnosis, past (e.g. “no shoes during childhood”) and present (e.g. self-perception of economic status) social disparities, and other covariables were analyzed.

**Results:**

From 8,848 older adults, 21.5% (*n* = 1903) were classified as prevalent cases (PG), 5.2% (*n* = 459) as incident cases (IG) and 77.4% (*n* = 6,486) were free of disease (NDG). The predictor variable “no shoes during childhood” was statistically significant in the model incident versus no diabetes group. Hypertension and body mass index (BMI) were the most relevant covariates as they were statistically significant in the three groups (PG, IG and NDG).

**Conclusions:**

Not having shoes during childhood, an indicator of social disadvantages, is associated with the incidence and prevalence of diabetes in older adults. This suggests that social disadvantages can be a determinant for the presence of chronic diseases in adulthood.

## Introduction

Poverty is a worldwide concern. In 2013, the World Bank estimated that 767 million people were living below the international poverty line of US$1.90 a day. This is equivalent to 10.7% of the total world population [1]. From these numbers, 186 million people lived in Latin America and the Caribbean [2]. Worldwide rates increase constantly, but in some countries, this rise is worrying. In Mexico, the poverty rate increased from 45.5 to 46.2% between 2012 and 2014 [3]. Older adults are particularly vulnerable to economic insecurity in both low- and high-income countries.

In 2015, rates of poverty in old age ranged from 3.2% in Denmark to 45.7% in Korea according to the Income Distribution Database of The Organization for Economic Co-operation and Development (OECD) [4]. It has been reported that in Latin America the poverty rates in older adults are lower than that observed in the younger population; however, the risk of remaining in this state is higher [5, 6]. According to the OECD report, 25.6% of older Mexican adults are in poverty [4] while national reports suggest that slightly above 25% of older adults live in this situation [7].

Recent theories about the influence of accumulated factors throughout life on the presence of diseases in the elderly have increased considerably. An example of this is the life course epidemiology framework [8–10] that addresses behavioral and psychosocial processes as factors that may exacerbate chronic disease risk during adulthood [11] such as overweight, obesity, and diabetes [12–14].

A possible explanation for the association between accumulated factors and chronic diseases is the early establishment of behavioral patterns, such as diet and exercise, or the appearance of metabolic changes associated with deprivation that may be related to patterns of morbidity in adult life [15]. An inverse association can also be proposed as the high costs associated with chronic disease may force adults into food insecurity [16].

Specifically about diabetes, there is limited understanding of the extent to which biological and social risks experienced at different stages of life combine to influence its presence in older adults, but plausible explanations have been reported. The early establishment of behavioral patterns, such as a low-quality diet and a sedentary lifestyle has been reported as possible mechanisms, and minority groups are especially vulnerable to adopting unhealthy behaviors [17, 18]. Furthermore, the appearance of an adaptive metabolic response associated with deprivation [15] and overcompensation during times of relative food adequacy, result in fast/binge cycles and have also been linked to insulin resistance [19].

In this context, this study aims to analyze whether the presence of social disadvantages in childhood and in the present affects the presence of diabetes in older adults.

## Materials and methods

The present study was based on longitudinal data from the third and fourth Mexican Health and Aging Study (MHAS) waves (2012 and 2015), which is a prospective panel study conducted in Mexico. The aim and design of the MHAS are published elsewhere [20, 21]. In brief, there are four waves of this study (2001, 2003, 2012, and 2015) with a representative sample of community-dwelling older Mexican adults. A set of questionnaires (socio-demographic, health-related, cognitive performance, functional status, among others) were applied by standardized interviewers, who were trained in the objectives of the survey, the questionnaires as well as the process of face-to-face interview. The complete questionnaires can be reviewed at the web page of the MHAS at http://mhasweb.org/index.aspx [21].

A total of 13,628 participants older than 50 years who were assessed in both waves, 2012 and 2015, were included. After excluding those < 50 years (*n* = 921), those with inconsistent answers to the self-reported diabetes status question (*n* = 286), and individuals with missing data in the independent variables and the covariables (*n* = 3,573), a final sample of 8,848 individuals was analyzed.

The main outcome was diabetes, which was considered to be present if the older adult answered “yes” to the question “Has a doctor or medical personnel (ever/in the last two years) diagnosed you with diabetes?” According to the diabetes status in both waves three groups were obtained: incident (reported diabetes in 2015), prevalent (reported diabetes since 2012), or without diabetes (no diabetes in both waves).

In order to assess factors associated with the incidence and prevalence of diabetes, a set of independent variables regarding social disadvantages was analyzed: disadvantages in childhood was requested through the questions “Before you were age 10, did you wear shoes or other footwear regularly?” and “Before you were age 10, generally, did you go to sleep hungry?”

Current disadvantages were analyzed through the question “Would you say your financial situation is...” and the right to health insurance inquired by the question “Do you have the right to medical attention in…?” Also, food shortage was evaluated through two questions: “In the last two years, have you always had sufficient money to buy the food that you need?” Those who answered “no” to the previous question also replied to the following “At any time in the last two years, did you not eat or eat less than you wanted because there was not enough food in your home?”

Covariables were selected based on the criteria of the researcher’s team. Variables from different dimensions were explored using the 2012 wave: socio-demographic characteristics included age, sex, marital status, and health services provider. Schooling was also included. The dimension of health-related variables included self-reported hypertension, cancer, heart attack, lung chronic disease (asthma or emphysema), and stroke. The mental health related variable was depression, measured through a nine-item questionnaire validated in the Mexican population. The cut-off point positive to depression was a score of 5 or higher [22]. Internal locus of control was also evaluated through the following questions: “One is responsible for his/her own successes”; “One can do just about anything he/she puts his/her mind to”; “One’s misfortunes are the result of his/her own mistakes” ; and “One is responsible for his/her own failures [23]” and, each one with a score from 1 to 4. Scores under the average means the presence of internal locus of control and higher scores, the absence of internal locus of control. Data on smoking and alcohol drinking habit were also collected.

Self-reported weight and height were used to calculate body mass index (BMI) (kg/m2) and categorized into underweight (< 22 kg/m2), normal (22.1–26.9 kg/m2), overweight (27–29.9 kg/m2), and obese (≥30 kg/m2) [24, 25]. Health status self-perception was also analyzed in two categories, good (excellent/very good/good) and bad (fair/poor), as described previously by other researchers [26, 27].

An activities of daily living (ADL) questionnaire was used to evaluate the functionality dimension. For the ADLs [28], participants were asked whether they needed help for walking around the house, bathing, using the toilet, and getting in/out of bed. The number of limitations present was also analyzed.

### Statistical analysis

Frequencies and percentages were calculated for the full sample and for each group. Based on the three groups obtained according to the diabetes status in both waves, two multiple logistic regressions were fitted: 1) a regression consisting of a dependent variable comparing the incident with the no diabetes group; and 2) a regression consisting of a dependent variable comparing the prevalent with the no diabetes group. Variables from different dimensions were selected according with the criteria of the researchers and excluded from the final model because they were not significant. All analyses were performed with the statistical package software STATA 14® [29].

### Ethical issues

The MHAS was approved by the Institutional Review Boards and Ethics Committees of the University of Texas Medical Branch in the USA, the Instituto Nacional de Estadística y Geografía (INEGI), and the Instituto Nacional de Salud Pública (INSP) in Mexico. The current analysis was registered at the Instituto Nacional de Geriatría (DI-PI-006/2018).

## Results

**Fig. 1:**
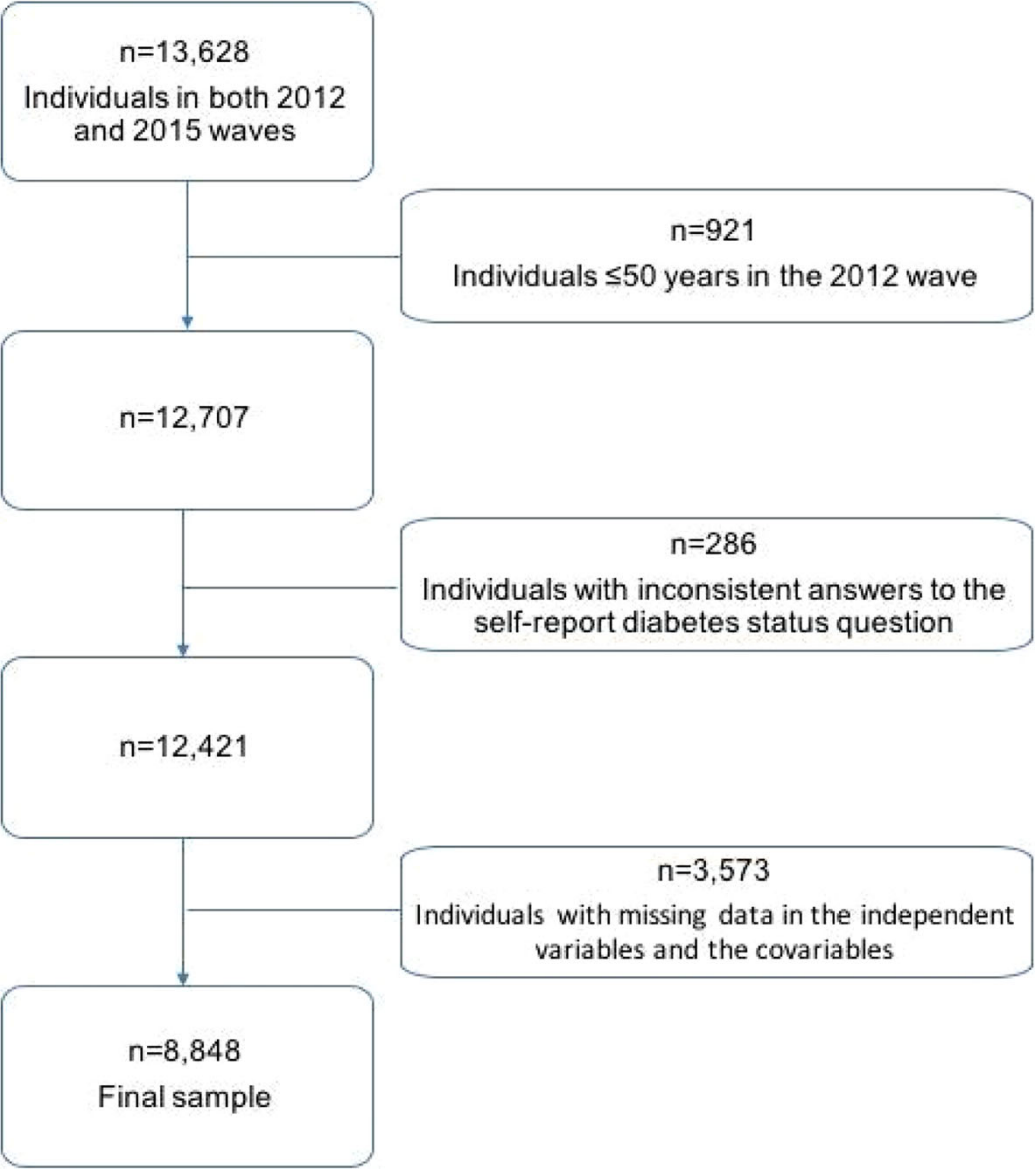
Flow of participants

A total of 8,848 participants were included in the analysis. The flow of participants can be seen in Fig. 1.

From the sample studied, 459 participants (5.19%) were classified in 2015 as incident cases (IG) and 1,903 (21.51%) as prevalent cases (PG). A total of 6,486 (73.30%) participants were free of disease (NDG) in 2012 and 2015.

Sociodemographic characteristics of the studied population are reported in Table 1. Mean age is similar between groups, 63.68 years old, except for the IG, which reports a slightly lower mean (62.69). Most of the participants were women and percentages range from 54.46% in the NDG to 62.01% in the PG. Regarding schooling, no differences were found either in the number of years of formal education (mean = 6.24 years) or in the percentage of participants without education (around 14% in the three groups). The percentage of individuals reporting bad self-perception of economic status was higher in the PG (80.82%) followed by the IG (75.96%). (Table 1).

**Table 1:** Sociodemographic characteristics of the studied sample

	Diabetes Incident Group (IG) *n* = 459	Diabetes Prevalent Group (PG) *n* = 1,903	No diabetes (NDG) *n* = 6,486	Total *n* = 8,848
Age, n(%)				
50–59	191 (41.61)	610 (32.05)	2,507 (38.65)	3,308 (37.39)
60–69	165 (35.95)	823 (43.25)	2,267 (34.95)	3,255 (36.79)
70–79	83 (18.08)	398 (20.91)	1,278 (19.70)	1,759 (19.88)
≥ 80	20 (4.36)	72 (3.78)	434 (6.69)	526 (5.94)
Mean, (SD)	62.69 (8.69)	63.95 (8.16)	63.67 (9.43)	63.68 (9.14)
Sex, n(%)				
Men	197 (42.92)	723 (37.99)	2,954 (45.54)	3,874 (43.78)
Women	262 (57.08)	1,180 (62.01)	3,532 (54.46)	4,974 (56.22)
Civil status, n(%)				
Single	20 (4.36)	71 (3.73)	316 (4.87)	407 (4.60)
Married/Civil Union	336 (73.20)	1,348 (70.84)	4,631 (71.40)	6,315 (71.37)
Divorced/Separated	36 (7.84)	135 (7.09)	531 (8.19)	702 (7.93)
Widowed	67 (14.60)	349 (18.35)	1,008 (15.54)	1,424 (16.09)
Schooling, n(%)				
No education	63 (13.73)	284 (14.92)	950 (14.65)	1,297 (14.66)
Incomplete primary school	152 (33.12)	604 (31.74)	1,817 (28.01)	2,573 (29.08)
Primary	99 (21.57)	520 (27.33)	1,641 (25.30)	2,260 (25.54)
Secondary	59 (12.85)	225 (11.82)	788 (12.15)	1,072 (12.12)
High school or higher	86 (18.74)	270 (14.19)	1,290 (19.89)	1,646 (18.60)
Mean (SD)	6.22 (5.06)	5.73 (4.51)	6.40 (5.03)	6.24 (4.92)
Self-perception of economic status, n(%)				
Bad	349 (76.03)	1,538 (80.82)	4,927 (75.96)	6,814 (77.01)
Good	110 (23.97)	365 (19.18)	1,559 (24.04)	2,034 (22.99)
Health Services Provider, n(%)				
Social Security Institutions	256 (55.77)	1,054 (55.39)	3,361 (51.82)	4,671 (52.79)
Ministry of Health	97 (21.13)	452 (23.75)	1,560 (24.05)	2,109 (23.84)
Private / Other	13 (2.83)	34 (1.79)	158 (2.44)	205 (2.32)
More than one service	41 (8.93)	231 (12.14)	628 (9.68)	900 (10.17)
Without provider	52 (11.33)	132 (6.94)	779 (12.01)	963 (10.88)

Regarding health services provider, most of the participants were insured by social security institutions and percentages range from 51.82% in the NDG to 55.77% in the IG. From the total sample, 10.88% reports not having a health service provider.

Clinical and psychological characteristics of the studied sample are presented in Table 2. Self-report of diagnosed hypertension, heart attack and stroke were higher in the PG (62.22, 5.62, and 3.05% respectively) followed by the IG (47.71, 3.70, and 1.96% respectively). Depression prevalence was found higher in the NDG (72.25%).

**Table 2:** Clinical and Psychological Characteristics of Sample studied

	Diabetes Incident Group *n* = 459	Diabetes Prevalent Group *n* = 1,903	No diabetes *n* = 6,486	Total *n* = 8,848
Hypertension, n(%)	219 (47.71)	1,184 (62.22)	2,501 (38.56)	3,904 (44.12)
Cancer, n(%)	9 (1.96)	53 (2.79)	118 (1.82)	180 (2.03)
Heart attack, n(%)	17 (3.70)	107 (5.62)	182 (2.81)	306 (3.46)
Lung Chronic Disease, n(%)	27 (5.88)	104 (5.47)	366 (5.64)	497 (5.62)
Stroke, n(%)	9 (1.96)	58 (3.05)	98 (1.51)	165 (1.86)
Depression, n(%)	316 (68.85)	1,192 (62.64)	4,686 (72.25)	6,194 (70.00)
Internal Locus of Control, n(%)	313 (68.19)	1,325 (69.63)	4,469 (68.90)	6,107 (69.02)
Mean (SD)	5.20 (1.64)	5.17 (1.71)	5.19 (1.71)	5.18 (1.71)
Smoking				
Never	299 (65.14)	1,273 (66.89)	3,968 (61.18)	5,540 (62.61)
Sometime / ex-smoker	104 (22.66)	465 (24.44)	1,609 (24.81)	2,178 (24.62)
Currently smoker	56 (12.20)	165 (8.67)	909 (14.01)	1,130 (12.77)
Alcohol drinking, (%)				
Never	51 (11.11)	249 (13.08)	742 (11.44)	1,042 (11.78)
Sometime	334 (72.77)	1,421 (74.67)	4,489 (69.21)	6,244 (70.57)
Yes, moderate	31 (6.75)	98 (5.15)	536 (8.26)	665 (7.52)
Yes, severe	43 (9.37)	135 (7.09)	719 (11.09)	897 (10.14)
Body Mass Index, n(%)				
Low weight	10 (2.18)	123 (6.46)	642 (9.90)	775 (8.76)
Normal	167 (36.38)	692 (36.36)	2,728 (42.06)	3,587 (40.54)
Overweight	114 (24.84)	458 (24.07)	1,526 (23.53)	2,098 (23.71)
Obesity	168 (36.60)	630 (33.11)	1,590 (24.51)	2,388 (26.99)
Mean (SD)	29.00 (4.73)	28.49 (5.01)	27.30 (4.59)	27.64 (4.73)
Health Self-perception, n(%)				
Bad	291 (63.40)	1,543 (81.08)	3,696 (56.98)	5,530 (62.50)
Good	168 (36.60)	360 (18.92)	2,790 (43.02)	3,318 (37.50)
Help walking around the house, n(%)	4 (0.87)	47 (2.47)	52 (0.80)	103 (1.16)
Help bathing, n(%)	2 (0.44)	39 (2.05)	49 (0.76)	90 (1.02)
Help eating, n(%)	2 (0.44)	35 (1.84)	29 (0.45)	66 (0.75)
Help using toilet, n(%)	3 (0.65)	37 (1.94)	41 (0.63)	81 (0.92)
Help getting in/out bed, n(%)	6 (1.31)	52 (2.73)	66 (1.02)	124 (1.40)
Number of limitations, ADL n(%)				
0	450 (98.04)	1,803 (94.75)	6,346 (97.84)	8,599 (97.19)
1	4 (0.87)	44 (2.31)	90 (1.39)	138 (1.56)
2	3 (0.65)	24 (1.26)	22 (0.34)	49 (0.55)
3	1 (0.22)	14 (0.74)	15 (0.23)	30 (0.34)
4	1 (0.22)	14 (0.74)	7 (0.11)	22 (0.25)
5	0 (0.00)	4 (0.21)	6 (0.09)	10 (0.11)

Percentages of participants with internal locus of control and history of never drinking alcohol were very similar among groups. The proportion of participants who reported never smoking was higher in the IG and PG (65.14 and 66.89%) compared with the NDG (61.18%).

As expected, percentages of overweight and obese participants were higher in the IG (24.84 and 36.60%), as well as in the PG (24.07 and 33.11%). Report of bad self-perception of health was more frequent in the PG (81.08%) and the IG (63.40%) compared to the NDG (56.98%).

With regard to functionality, percentages of disability were very low. The higher percentages were found in the PG for all types of ADL. Also, the percentage of individuals with one or more limitations was higher in the PG (5.26%) compared with the IG (1.96%) and the NDG (2.16%).

Concerning the social determinants, a higher percentage of individuals that reports no shoes during childhood was found in the IG (26.80%). Smaller percentages were reported in the PG (20.13%) and the NDG (21.03%). Went to bed hungry, not having enough money to buy food during the last 2 years, and household food shortage percentages were similar between groups. (Table 3).

**Table 3:** Social determinants

	Diabetes Incident Group (IG) *n* = 459	Diabetes Prevalent Group (PG) *n* = 1,903	No diabetes (NDG) *n* = 6,486	Total *n* = 8,848
No shoes during childhood, n (%)	123 (26.80)	383 (20.13)	1,364 (21.03)	1,870 (21.13)
Went to bed hungry before 10 years, n (%)	137 (29.85)	606 (31.84)	1,857 (28.63)	2,600 (29.39)
Not enough money to buy food in the last 2 years	150 (32.68)	649 (34.10)	2,065 (31.84)	2,864 (32.37)
Household’s food shortage /scarce food	68 (45.33)	303 (46.69)	942 (45.62)	1,313 (45.84)

The odds ratios associated with a logistic regression that compare the incident (success) with the no diabetes group (failure) are presented in Table 4, first column. The full logistic regression model containing all six main predictors and all the covariates was statistically significant (χ ^2^ = 99.50, df = 38, *n* = 6945, *p* < 0.01). Only the predictor variable “no shoes during childhood” was statistically significant (OR = 1.47, 95%CI 1.16–1.86, *p* < 0.01). For BMI, a person with normal weight is about 3.8 times as likely to be an incident instead of a no diabetes case than a person with low weight, controlling for the other predictors in the model. Similarly, for people with overweight and obesity (odds ratios of almost five and six, respectively). People with hypertension tend to be an incident instead of a no diabetes case (OR = 1.30, 95%CI = 1.06–1.59, *p* = 0.01).

**Table 4:** Logistic regressions comparing incident, prevalent and no diabetes cases groups^a^

Variable	Incidence group versus No Diabetes Group				Prevalence versus No Diabetes Group			
	OR	*p*-value^c^	95% CI		OR	*p*-value^c^	95% CI	
			LL	UL			LL	UL
Group of Age (ref. 50–59)^b^		**< 0.01**				0.36		
60–69	0.91	0.40	0.72	1.14	1.27	**< 0.01**	1.12	1.45
70–79	0.82	0.19	0.62	1.10	0.96	0.63	0.82	1.13
80 and more	0.67	0.12	0.40	1.11	0.49	**< 0.01**	0.37	0.66
Woman	0.92	0.51	0.73	1.17	0.99	0.89	0.87	1.13
Without partner	0.99	0.93	0.79	1.25	1.03	0.67	0.91	1.17
Years of formal education	0.99	0.59	0.97	1.02	0.99	0.40	0.98	1.01
Bad self-perception of economic status	0.89	0.36	0.69	1.14	0.92	0.29	0.80	1.07
Health services provider (ref. Social security)^b^		**< 0.01**				0.43		
Ministry of Health	0.79	0.07	0.61	1.02	0.90	0.14	0.78	1.03
Private / Other	1.12	0.71	0.62	2.01	0.83	0.35	0.56	1.23
More than one service	0.87	0.42	0.62	1.23	1.21	**0.03**	1.02	1.45
Without provider	0.89	0.49	0.65	1.23	0.61	**< 0.01**	0.49	0.75
Hypertension	1.30	**0.01**	1.06	1.59	1.96	**< 0.01**	1.75	2.20
Cancer	0.94	0.86	0.47	1.88	1.20	0.30	0.85	1.70
Heart Attack	1.16	0.57	0.69	1.96	1.40	**0.01**	1.08	1.82
Respiratory Failure	0.94	0.76	0.62	1.41	0.71	**< 0.01**	0.56	0.90
Stroke	1.13	0.73	0.56	2.30	1.21	0.30	0.85	1.72
Depression	1.06	0.59	0.85	1.33	1.06	0.36	0.94	1.20
No internal locus of control	1.05	0.64	0.85	1.29	1.00	0.93	0.88	1.12
Smoking (ref. never smoke)^b^		**0.02**				0.58		
Sometime	0.87	0.29	0.68	1.12	0.97	0.71	0.85	1.12
Current smoker	0.95	0.74	0.69	1.30	0.75	**< 0.01**	0.62	0.91
Alcohol Drinking (ref. never drink)^b^		**< 0.01**				0.52		
Sometime	1.11	0.51	0.81	1.52	1.03	0.75	0.87	1.22
Yes, moderate	0.91	0.70	0.56	1.48	0.75	**0.05**	0.57	1.00
Yes, severe	0.91	0.70	0.58	1.44	0.73	**0.02**	0.56	0.94
Body Mass Index (ref. undernutrition)^b^		**< 0.01**				**< 0.01**		
Normal weight	3.83	**< 0.01**	2.01	7.32	1.33	**0.01**	1.07	1.66
Overweight	4.47	**< 0.01**	2.32	8.64	1.41	**< 0.01**	1.12	1.78
Obesity	6.07	**< 0.01**	3.16	11.64	1.63	**< 0.01**	1.30	2.05
Bad health self-perception	1.21	0.10	0.96	1.51	2.67	**< 0.01**	2.33	3.07
Help walking around the house	1.47	0.53	0.44	4.91	1.79	**0.03**	1.06	3.01
Help bathing	0.46	0.36	0.09	2.40	1.13	0.70	0.61	2.08
Help eating	1.09	0.91	0.23	5.23	2.50	**< 0.01**	1.39	4.49
Help using toilet	0.99	0.99	0.25	4.03	1.03	0.93	0.54	1.97
Help getting in/out bed	1.18	0.74	0.45	3.10	1.30	0.27	0.82	2.08
No shoes during childhood	1.47	**< 0.01**	1.16	1.86	0.88	0.07	0.76	1.01
Went to bed hungry before 10 y	0.97	0.81	0.77	1.22	1.11	0.12	0.98	1.26
Not enough money to buy food in the last 2 years	1.05	0.71	0.81	1.36	1.00	0.98	0.86	1.15
Household’s food shortage /scarce food	1.03	0.84	0.77	1.38	1.00	0.99	0.85	1.17

^a^OR Odds Ratio; CI Confidence Interval; LL Lower Limit; UL Upper Limit

^b^Wald test for categorical variables, χ^2^ with number of categories less 1 degrees of freedom

^c^Significant p values (*p* < 0.05) are in bold

Finally, the odds ratios associated with a logistic regression that allows us to compare the prevalent (success) with the no diabetes group (failure) are presented in Table 4, second column. It can be observed that the full logistic regression model containing all six main predictors and all the covariates was statistically significant (χ^2^ = 820.71, df = 38, *n* = 8389, *p* < 0.01). Only health services provider was statistically significant. Thus, first when holding all the other predictors constant, a person covered by more than one health service is 1.21 times more likely to be a prevalent instead of a no diabetes case than a person affiliated to social security institutions. Second, a person without medical services is 0.61 times more likely to be a prevalent instead of a no diabetes case than a person affiliated to social security institutions. The odds ratio for age indicates that patients between 60 to 69 years old are 1.27 times more likely to be a prevalent instead of a no diabetes case than those between 50 and 60 years old. On the other hand, people aged 80 years or more are 0.49 times more likely to be a prevalent instead of a no diabetes case than people in the reference group. For BMI, a person with normal weight is about 1.33 times more likely to be a prevalent instead of a no diabetes case than a person with low weight, controlling for the other predictors in the model. The results are similar for overweight and obese people. Current smokers are 0.75 times more likely to be a prevalent instead of a no diabetes case than those who had never smoked. A person that drinks in a moderate or severe way is 0.75 and 0.73, respectively, more likely to be a prevalent instead of a no diabetes case than a person who did not drink alcohol. People with bad health self-perception are 2.67 times more likely to be a prevalent instead of a no diabetes case than those persons with good health condition, controlling for the other predictors. History of hypertension or heart attack tend to be prevalent instead of no diabetes cases, but participants without lung chronic disease have lower odds of being prevalent case compared to NDG in 29% (OR = 0.71, 95% CI = 0.56–0.90, *p* < 0.01). Also, getting help walking around the house or eating makes people more prone to diabetes.

## Discussion

This study presents the results of the relationship between social disadvantages during childhood and at the present, and the incidence and prevalence of diabetes in a national representative sample of older adults. Due to the longitudinal design of the MHAS, we were able to examine how social disadvantages during childhood and adulthood influence the likelihood of diabetes outcome. Consequently, it appears possible to elucidate whether either adulthood factors or early life socioeconomic conditions are the most critical outcomes associated with having diabetes.

The findings from this study are partially congruent with other studies [30–33]. The only social determinant that was significant to the incident cases versus NDG was “no shoes during childhood”. The other indicator of childhood poverty, “went to bed hungry before 10” was not found associated. From the descriptive analysis, we found similar proportions of these two variables between the groups. “No shoes during childhood” is a widely used indicator of child poverty [34, 35]. Specifically, it has been reported as part of the basket of non-food items [36] and used by various international organizations as an indicator of effective satisfaction of needs, reflecting not only economic but social deprivation [37]. On the other hand, it has been reported that child poverty is strongly related to the presence of diabetes later in life [38, 39]. Then, it may be possible that the indicator “no shoes during childhood” could be a stronger indicator of social disadvantages, poverty and a predictor to diabetes incidence. Against expectation only this indicator was significant. A reason behind could be that “no shoes during childhood” is a more objective indicator not affected by the possible memory bias of participants.

We further found that social constraints at early stages in life can lead to a chronic lack of resources and are linked with the presence of chronic disease such as overweight, obesity, and diabetes during adulthood [12–14]. It is well known that the choices made by children and adolescents are strongly affected by the family and community environments in which they live. But also, impoverished living conditions prevent people from engaging in healthy behaviors [40]. How is poverty linked to obesity and diabetes? [41]. It has been suggested that poor families choose high fat, sugar, and sodium foods because these foods are more affordable, convenient, and last longer than fresh vegetables, fruits, and lean meats and fish [42]. Low-income families often live in disadvantaged neighborhoods where healthy foods are hard to find [43–46]. But also, economic problems such as troubles paying the rent or bills stress people and they often cope by eating high-fat, or sugary foods [47]. On the other hand, individuals who live in impoverished regions have reduced access to parks or gyms for regular physical activity. In many poor neighborhoods, parks, free public gyms are often not available or safe [48–51].

A result that consistently was present along the three groups (IG, PG, NDG) was the body mass index. An increased and statistically significant odds ratio was present not only for overweight and obese participants but also normal weight group versus low weight individual. This finding represents not only a clear and strong relationship between weight and diabetes [52], but also it could mean that the cutoff point could be biased towards the underestimation of risk. It is important to highlight that the reference values used for the BMI in the present analysis were those proposed by Lipschitz [25, 53]. These cutoff points consider that in older adults, weight tends to decrease due to a reduction in body water and muscle mass. If the traditional BMI classification for adults [54] would have been used, the individual risk for diabetes would be lower for those classified as normal weight and higher for those in the overweight and obesity groups. Nevertheless, the direction of the association shows that the risk of developing diabetes increases with a higher BMI.

The evidence is more robust for the long-term influence of early life vulnerable socioeconomic conditions on the development of diabetes [13, 32] during adulthood. In our study current disadvantages do not appear to contribute more to the association than social disparities during childhood. However, other variables such as age, health services, BMI, and health perception overshadowed the association between early life socioeconomic conditions and diabetes. The presence of adverse socioeconomic conditions in the early stages of life have a significant potential impact on health at older ages [55].

When the incident group was compared to the no diabetes group, having hypertension seems to be associated with the occurrence of diabetes (OR = 1.30, 95%CI 1.06–1.59, *p* = 0.01). This association was expected as concomitant diabetes and hypertension is a frequent condition in older adults [56, 57].

In our sample of prevalent cases against no diabetes cases, we found that having more than one health service increases the risk of being a prevalent case. Why might the pathway via insurance coverage be so critical? Before considering possible explanations we need to consider whether this finding could be because of *self*-*report* response *bias*. Since the analysis is based on self-reported data, the data from the Mexican Health and Aging Study may underestimate the prevalence of diabetes. Thus, the association between affiliation to social security institutions and prevalence of diabetes needs further study. When we compare PG versus NDG, the figure indicates that it is a protective factor, and it can be seen as an indicator of diagnosis delay [58]. In this regard, it is worth noting that not having received the diagnosis does not mean the absence of disease. Also, some differences between IG and PG could be explained by clinical course and time living with the disease, a process of adaptation to the disease and survival could also be a reason of differences between PG and IG.

This study has several limitations. The measurement of childhood conditions can be subject to recall bias. In this respect, previous reports support the ability of people in recalling early-life events and childhood health conditions [59], so that information can be considered a good approach to the early-life information in this sample of older adults. Also, considering that objective measurements along the life course are difficult, retrospective reports are a reliable source of information.

For the self-reported diabetes condition, this recall bias might have affected our findings regarding the real presence of the diabetes diagnosis and its predictors. In this sense, it has been reported that prevalent self-reported diabetes and incident self-reported diabetes has 84–97% specificity and 55–80% sensitivity in respect to multiple reference definitions. Also, the reliability of self-reported diabetes is > 92% at all time points [60]. In this sense, our results can be considered an accurate approximation to the presence of diabetes in older Mexican adults.

Taking into account that the “no shoes during childhood” variable is not an indicator of childhood poverty commonly used in other research, comparisons with other populations may be difficult. Notwithstanding, this indicator confers a novelty to our analysis as it has demonstrated to be a reliable indicator of childhood poverty and a good predictor of diabetes, a highly prevalent chronic disease in older adults. However, we suggest that a single indicator need to be used with caution as poverty is a more complex phenomenon.

## Data Availability

Data of this research is available with the correspondant author (CGP).

## Abbreviations

ADL: Activities of daily living
BMI: Body mass index
CI: Confidence Interval
df: Degrees of freedom
IG: Incident cases
INEGI: Instituto Nacional de Estadística y Geografía
INSP: Instituto Nacional de Salud Pública
MHAS: Mexican Health and Aging Study
NDG: Free of disease
OECD: Organization for Economic Co-operation and Development
OR: Odds ratio
PG: Prevalent cases
